# Development of icterus gravis in a preterm infant with G71R UGT1A1 polymorphism

**DOI:** 10.1186/1756-0500-6-51

**Published:** 2013-02-06

**Authors:** Akimune Kaga, Yukimune Ohkubo, Yohei Watanabe, Sachiko Saito, Takuma Matsuki, Haruo Usuda, Susumu Kanda, Yutaka Suzuki, Muneyuki Tanabu, Shigeo Kure

**Affiliations:** 1 Department of Pediatrics, Hachinohe City Hospital, 1 Bisyamontaira, Hachinohe, Aomori, 031-8555, Japan; 2 Department of Pediatrics, Tohoku University School of Medicine 1-1, Seiryo-machi, Aoba-ku, Sendai, Miyagi, 980-8574, Japan

**Keywords:** Gilbert’s syndrome, G71R, Icterus gravis, Preterm infant, Uridine diphosphate-glucuronosyltransferase

## Abstract

**Background:**

Uridine diphosphate-glucuronosyltransferase (UGT) gene family is involved in the detoxification of biomaterials and drugs in the liver. Among the UGT gene family members, only UGT1A1 is involved in bilirubin conjugation. As a result, deficient UGT1A1 activity causes jaundice. One disease that is characterized by reduced UGT1A1 activity is Gilbert’s syndrome. Two prevalent UGT1A1 polymorphisms responsible for Gilbert’s syndrome have been identified: G71R in exon 1 and A(TA)7TAA in the TATA box of the promoter region. Recently, the G71R polymorphism has been associated with breastfeeding jaundice and neonatal hyperbilirubinemia in term infants. However, its association with jaundice in very low birth weight infants (VLBWIs) has never been reported.

**Case presentation:**

The patient was a female born at 28 weeks, 4 days gestation with a birth weight of 1172 g. On day 21, intense yellowing of the skin and eyes was noted, and the patient’s total bilirubin level was 23.7 mg/dL (her direct bilirubin level was 2.1 mg/dL). Therefore, an exchange transfusion was conducted. She had neither blood type incompatibility nor a family history of constitutional jaundice. Metabolic screens for amino and organic acids were negative. No elevation of any of the examined antibody titers was noted, and no evidence of an inflammatory reaction was observed. In addition, no hematological abnormalities were detected. The direct/indirect Coombs test, irregular antibody test and red blood cell antibody dissociation test were all negative, and her thyroid function was normal. We performed sequence analysis of the UGT1A1 gene after the patient’s parents provided written informed consent. Exon 1 of the UGT1 gene on chromosome 2 was analyzed by direct sequencing. A heterozygous substitution from G to A (211G→A: G71R) in base 211 was noted.

**Conclusion:**

We speculated that this preterm infant with carrying the G71R polymorphism reduced UGT1A1 activity and developed severe jaundice that was likely triggered by factors such as breast feeding and medications. The polymorphism appears at some frequency among VLBWIs, which would necessitate adequate care of severe jaundice even after the acute phase.

## Background

Uridine diphosphate-glucuronosyltransferase (UGT) gene family is involved in the detoxification of biomaterials and drugs in the liver. Among the UGT gene family members, only UGT1A1 is involved in bilirubin conjugation. As a result, deficient UGT1A1 activity causes jaundice. One disease that is characterized by reduced UGT1A1 activity is Gilbert’s syndrome. Two prevalent UGT1A1 polymorphisms responsible for Gilbert’s syndrome have been identified: G71R in exon 1 and A(TA)7TAA in the TATA box of the promoter region. Homozygous and compound heterozygous mutations of these genetic regions can result in severe jaundice, and heterozygous mutations serve as a risk factor for the development of jaundice [1]. Recently, the G71R polymorphism has been associated with breastfeeding jaundice and neonatal hyperbilirubinemia in term infants [2-4]. However, its association with jaundice in very low birth weight infants (VLBWIs) has never been reported. In this article, we report case study of a VLBWIs carrying the G71R polymorphism who presented with severe jaundice that was first noticed on day 21 after birth.

## Case presentation

The patient was a female born at 28 weeks, 4 days gestation with a birth weight of 1172 g. The mother was 29 years old with a history of 1 prior pregnancy, which ended in a spontaneous abortion. After 28 weeks and 3 days of pregnancy, the mother was hospitalized because of genital bleeding and labor pains. The labor pains were difficult to suppress, and the mother delivered a baby transvaginally the following day. When the placenta was checked macroscopically, the chorion and umbilical cord facing the fetus were found to be edematous and yellow. Pathologically, mild neutrophil infiltration of the amniotic membrane stroma was noted and was accompanied by necrosis of the umbilical cord stroma and the presence of degenerative neutrophils.

**Figure 1:**
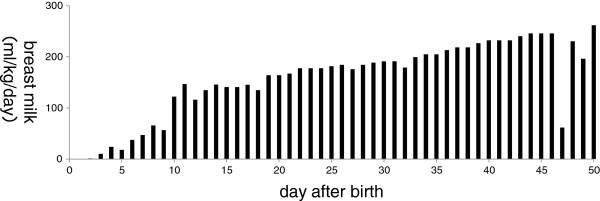
**Amount of breast milk.** Breastfeeding commenced on day 3, and exclusive breastfeeding was achieved on day 10.

The patient was intubated at birth and had Apgar scores of 6 at 1 minute and 7 at 5 minutes. She received surfactant on the day of birth. Elevated levels of IgM in the umbilical blood (35 mg/dL) indicated an infection, and the patient was administered antibiotics for 5 days. Indomethacin administration led to the closure of a patent ductus arteriosus by the second day of life, and tube feeding (artificial milk) was initiated one day after birth. Breastfeeding commenced on day 3, and exclusive breastfeeding was achieved on day 10 (Figure 1).

**Figure 2:**
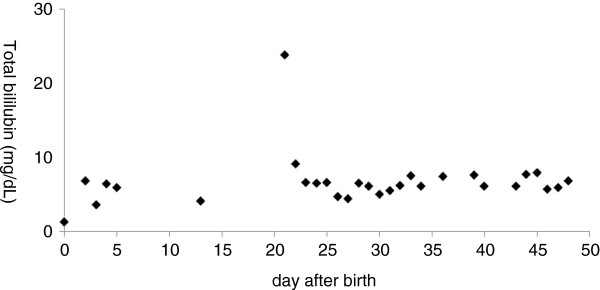
**Clinical course of jaundice.** On day 21, the total bilirubin level was 23.7 mg/dL (the direct bilirubin level was 2.1 mg/dL), and the exchange transfusion was performed.

The patient’s total bilirubin (TB) level was 6.8 mg/dL on day 2, and phototherapy was initiated for 12 hours. On day 21, intense yellowing of the skin and eyes was noted, and the patient’s TB level was 23.7 mg/dL (her direct bilirubin level was 2.1 mg/dL) (Figure 2). Therefore, an exchange transfusion was conducted in accordance with the criteria reported by Kawase et al. [5]. She had neither blood type incompatibility nor a family history of constitutional jaundice. Metabolic screens for amino and organic acids were negative. No elevation of any of the examined antibody titers was noted, and no evidence of an inflammatory reaction was observed. In addition, no hematological abnormalities were detected. The direct/indirect Coombs test, irregular antibody test and red blood cell antibody dissociation test were all negative, and her thyroid function was normal. No typical bilirubin encephalopathy symptoms were observed, and serial head ultrasonography revealed no lesions during hospitalization. The follow-up head magnetic resonance imaging was normal at 2 months of age, and her auditory brainstem responses were normal at 5 months of age. Within 1 year, the patient could walk and showed favorable growth and development.

**Figure 3:**
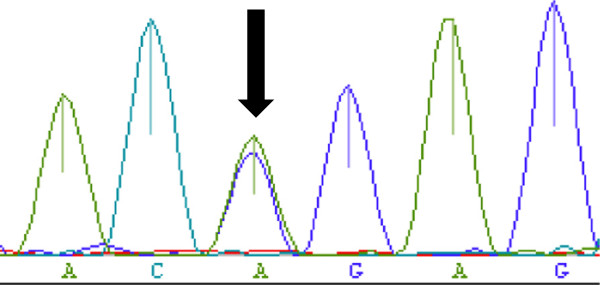
**UGT1A1 gene analysis.** A substitution at base 211 from G to A (211G→A: G71R) in exon 1 of the bilirubin uridine diphosphate-glucuronosyltransferase (UGT1A1) gene was observed with a heterozygous pattern (↓).

We performed sequence analysis of the UGT1A1 gene after the patient’s parents provided written informed consent. Exon 1 of the UGT1 gene on chromosome 2 was analyzed by direct sequencing. A heterozygous substitution from G to A (211G→A: G71R) in base 211 was noted (Figure 3).

## Discussion

The G71R polymorphism, which is common in East Asia, is a risk factor for hyperbilirubinemia [1,2] and has been associated with an approximate 40% reduction of UGT1A1 activity [6]. In term infants, UGT1A1 activity has been reported to be approximately 1% of that observed in adult values [7]. We speculated that the UGT1A1 activity in the case of the infant reported here (a VLBWI carrying the G71R polymorphism) was less than 1% of the typical activity in term infants. This infant with low UGT1A1 activity developed severe jaundice that was likely triggered by factors such as breast feeding [8] and medications.

Pregnanediol, which is present in maternal milk, is known to reduce UGT1A1 activity [8]. In the current case, the patient developed severe jaundice when her consumption of maternal milk increased after the age of 3 weeks (Figure 1).

A pathological examination of the placenta revealed slight neutrophil infiltration of the amniotic membrane stroma, accompanied by umbilical cord stroma necrosis, the appearance of degenerative neutrophils and elevated umbilical blood levels of IgM, findings consistent with intrauterine infection. Although severe jaundice developed on day 21 after birth in this infant, the involvement of infection in the development of jaundice could not be completely ruled out in this case.

The jaundice was severe enough to necessitate exchange transfusion. However, the patient never showed the classical symptoms of nuclear jaundice (reduced muscular tone, opisthotonus and spastic symptoms) [9,10]. The head magnetic resonance imaging, which was performed on day 60 after birth, revealed no abnormalities in any region of the brain, including the pallidum [10]. The auditory brainstem responses test conducted on day 172 after birth revealed bilateral responses and appropriate responses to sounds. Thus, the patient exhibited favorable growth and development. Although the patient has shown no signs of kernicterus to date, we propose to continue careful follow-up [10].

The estimated frequency of the G71R gene polymorphism in Japan is 0.15, and Gilbert’s syndrome affects 3-7% of the entire population. The polymorphism appears at a similar frequency among VLBWIs, which would necessitate adequate care of severe jaundice even after the acute phase. This UGT1A1 mutation is known to be related to diverse diseases and to elevate the risk of jaundice and gallstones [4]. Because treatment with several anti-cancer drugs (such as irinotecan hydrochloride) can cause bone marrow suppression and severe diarrhea in individuals with the G71R polymorphism, screening for polymorphisms in this gene prior to starting such treatments is authorized. In Japanese individuals with unexplained jaundice, a potential UGT1A1 mutation should be considered in the differential diagnosis, and preterm infants with this polymorphism may be at risk for late onset hyperbilirubinemia.

## Conclusion

We speculated that this preterm infant with carrying the G71R polymorphism reduced UGT1A1 activity and developed severe jaundice that was likely triggered by factors such as breast feeding and medications. The polymorphism appears at some frequency among VLBWIs, which would necessitate adequate care of severe jaundice even after the acute phase.

## Consent

Written informed consent was obtained from the patient’s guardian for publication of this study and accompanying images. A copy of the written consent is available for review by the Editor-in-Chief of this journal.

## Abbreviations

UGT: Uridine diphosphate-glucuronosyltransferase; VLBWIs: Very low birth weight infants; TB: Total bilirubin.

## Competing interests

The authors have no competing interests.

## Authors’ contributions

AK, YO, YW, SS, TM, SK, YS, and MT treated the patient. The patient was followed up by AK and UH. AK, YO, YW, SS, TM, HU, SK, YS, MT, and SK were involved with drafting of the manuscript. All of the authors have read and approved the final manuscript.
